# Application of a Novel PM Model to Assess the Risk of *Clostridioides difficile* Infections in Medical Facilities as a Tool for Improving the Quality of Health Services and the Safety of Patients

**DOI:** 10.3390/ijerph19010441

**Published:** 2021-12-31

**Authors:** Zofia Maria Kiersnowska, Ewelina Lemiech-Mirowska, Aleksandra Sierocka, Michał Zawadzki, Michał Michałkiewicz, Michał Marczak

**Affiliations:** 1 Department of Management and Logistics in Healthcare, Medical University of Lodz, 90-419 Lodz, Poland; ewelina.l.mirowska@wihe.pl (E.L.-M.); adreslewska@wp.pl (A.S.); michal.marczak@umed.lodz.pl (M.M.); 2 Laboratory of Epidemiology, Military Institute of Hygiene and Epidemiology (WIHE), 01-163 Warsaw, Poland; 3 Warsaw School of Information Technology (WIT) under the Auspices of the Polish Academy of Sciences, 01-447 Warsaw, Poland; michal.misiowiec@gmail.com; 4 Faculty of Environmental Engineering and Energy, Institute of Environmental Engineering and Building Installations, Poznan University of Technology, 60-965 Poznan, Poland; michal.michalkiewicz@put.poznan.pl

**Keywords:** Proposed Model (PM model), *Clostridioides difficile*, CDI, infection risk management

## Abstract

Infections with multi-drug resistant microorganisms associated with the provision of health services have become an acute problem worldwide. These infections cause increased morbidity as well as mortality and are a financial burden for the healthcare system. Effective risk management can reduce the spread of infections and thus minimize their number in hospitalized patients. We have developed a new approach to the analysis of hazards and of exposure to the risk of adverse events by linking the patient’s health record system to the entire infrastructure of the hospital unit. In this study, using the developed model, we focused on infections caused by the *Clostridioides difficile* bacterium, as they constitute a significant number of nosocomial infections in Poland and worldwide. The study was conducted in a medical facility located in the central part of Poland which provides tertiary care. In the proposed PM model, a risk analysis of hospital acquired infections at the Intensive Care and Anesthesiology Unit combined with the hospital’s technical facilities and organizational factors was conducted. The obtained results indicate the most critical events which may have an impact on potential hazards or risks which may result from the patient’s stay at the specific ward. Our method can be combined with an anti-problem approach, which minimizes the critical level of infection in order to determine the optimal functioning of the entire hospital unit. Research has shown that in most situations the spread dynamics of nosocomial infections can be controlled and their elimination may be attempted. In order to meet these conditions, the persons responsible for the daily operation of the medical facility and its individual wards have to indicate potential events and factors which present a risk to the hospitalized patients. On the basis of a created spreadsheet directions for improvement may be finally established for all potential events, their frequency may be minimized, and information may be obtained on actions which should be undertaken in a crisis situation caused by the occurrence of a given phenomenon. We believe that the proposed method is effective in terms of risk reduction, which is important for preventing the transmission of multi-drug resistant microorganisms in the hospital environment.

## 1. Introduction

A medical facility’s risk management strategy applied in emergency situations (which cannot always be predicted in advance, but cause serious consequences once they occur) usually proves to correct. The response of a medical facility to an equipment breakdown or to an adverse event and further actions taken by the facility, both on a macro and micro scale (within a given hospital), should be thoroughly investigated, analyzed and ultimately minimized by overcoming barriers to improved safety, barriers which may be caused by management style and culture, system, communication and organization of management processes.

Any acts of negligence on part of the hospital, absence of proper, systemic and integrative management, or various risk management processes may endanger the health and life of patients and result in a negative impact on the hospital’s medical quality, due to a reduced bed turnover rate, increased costs of treatment and financial losses for both the hospitalized persons and for the medical facility itself.

**Figure 1:**
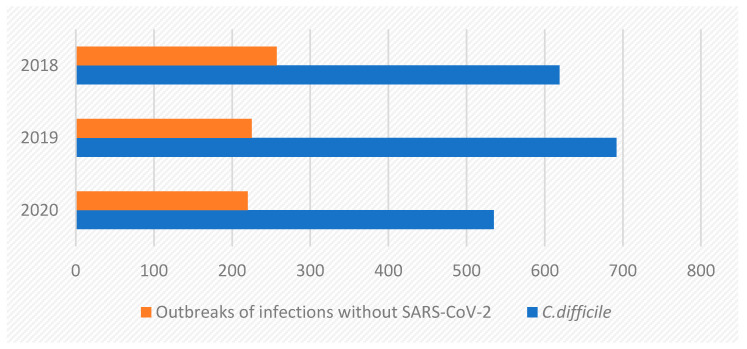
*C. difficile* outbreaks in Poland in 2018–2020 (Source: Chief Sanitary Inspector [11]).

Healthcare Associated Infections (HAI) caused mainly by multi-drug resistant microorganisms are currently a global problem which affects up to 25% of hospitalized patients in developing countries [1,2,3,4]. HAI affect primarily the respiratory system, the urinary tract, surgical sites and the gastrointestinal tract, in which infections are primarily caused by the *Clostridioides difficile* (*C. difficile*) bacterium. Apart from *C. difficile,* the most frequently mentioned microorganisms that pose a serious risk to the health and life of hospitalized patients include: *Salmonella* spp., *Escherichia coli*, *Campylobacter* spp., *Yersinia enterocolitica*, *Streptococcus* spp., *Staphylococcus aureus*, *Enterococcus faecium*, *Enterobacter cloacae*, *Klebsiella pneumoniae*, *Pseudomonas aeruginosa*, *Acinetobacter baumanii*, *Haemophiles influenzae*, *Mycoplasma pneumoniae*, and *Mycobacterium tuberculosis* [5,6,7,8]. Both Gram-positive and Gram-negative bacteria may develop antibiotic resistance mechanisms. In the hospital environment, a *C. difficile* infection is considered to be one of the most serious consequences related to the provision of health services [3,9,10]. In Poland in 2020 *C. difficile* was the cause of 200 out of 535 outbreaks, which accounted for 41.1% of registered infections (without SARS-CoV-2) (Figure 1) [11]. Symptoms of infection with this bacterium range from mild diarrhea and abdominal discomfort to more severe colitis and death. It is assumed that limiting the transmission of *Clostridium difficile* infections (CDI) is one of the major challenges facing inpatient treatment.

Recognizing the possibilities of occurrence of nosocomial infections, individual medical facilities should strive to develop and implement procedures for dealing with specific crisis situations. There are many methods of prevention, starting from regular infection risk assessments with the use of specialized tools and models, through periodical training, increased control over procedures, controlling of trends and direction of changes and ending with the use of the simplest and effective method, that is following correct hand hygiene procedures. Additionally, every case of nosocomial infection must be precisely diagnosed in the laboratory, in order to rapidly undertake appropriate method of treatment and to look for possible causes (sources) of the patient’s symptoms. Their introduction and maintenance are supervised by designated managers and hospital infection control teams. The literature shows that infection most often occurs in several categories of issues (hazards) [5,6,12,13,14,15]. These include both factors related to the admitted patient and those resulting from the functioning of the medical facility.

Due to the potentially emerging risk factors, we believe that it is very important to improve risk management in hospital units. For this purpose, an attempt was carried out to develop a novel functional model which supports the operation of a medical facility and responds to potential hazards. At the same time, it aims to identify the hazard risk and exposure to the risk of infection in real time and to indicate a way to improve the situation.

## 2. Materials and Methods

### 2.1. Model Characteristics

The proposed conceptual model (PM, short for Proposed Model) is used to recognize phenomena which may occur in any medical facility and their causes, while the results obtained from the analysis enable us to generate solutions intended to prevent the problem from reoccurring or to minimize it to an acceptable level. The model was developed by our team in order to create a useful quality support tool that introduces improvements in the functioning of a hospital, which can be viewed as a process or an organization. The idea behind the PM is to increase the level of patient safety as well as the safety of medical and auxiliary staff. The new PM model adapted to the conditions in hospitals in Poland was created based on the guidelines and the model created by the Association for Professionals in Infection Control and Epidemiology (APIC) [16,17,18].

**Figure 2:**
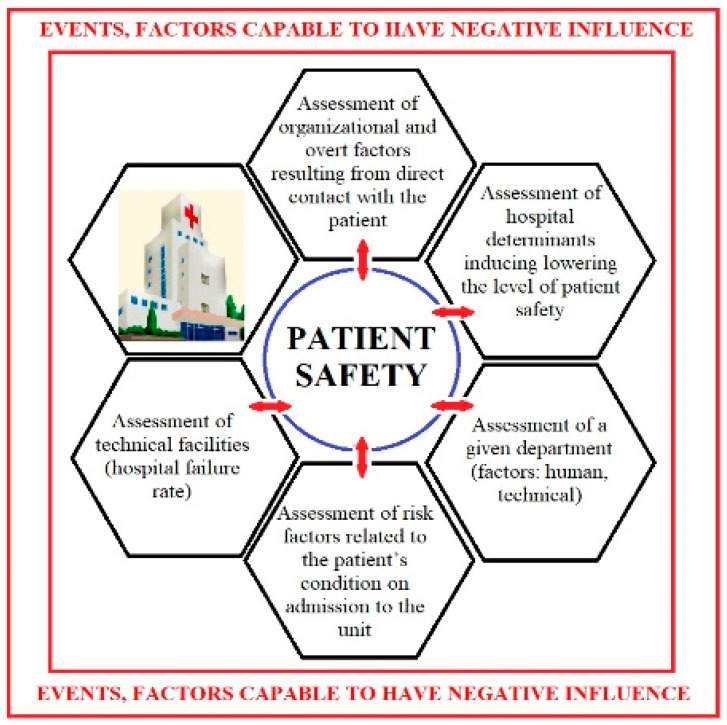
A competency model for predicting the risk of hazards and exposure to the risk of nosocomial infections.

The essence of the PM is a clear, graphical presentation of relative hazard areas, and then the development of individual categories of problems in order to get to the error’s root cause and to indicate a possible means of preventing it. This conceptual model is presented in the form of a chart (Figure 2). Patient safety is at the center of the diagram. The aspiration goal of the model consists of all elements originating from the main core of the diagram to the outside, i.e., the categories of issues which have an impact on the level of patient safety in accordance with the PM’s concept. These include categories such as: Assessment of technical facilities (hospital failure rate); Assessment of organizational factors; Assessment of overt factors resulting from direct contact with the patient; Assessment of a given department (factors: human, technical); Assessment of risk factors related to the patient’s condition upon admission to the unit (and during their stay in the hospital) that may have a negative impact on the safety of the patient, contact patients, staff and the entire hospital environment; and Assessment of hospital determinants which decrease of the safety level of a patient undergoing hospitalization.

Each of the hazard categories is subject to individual, comprehensive assessment of risk and of exposure to the risk of HAI. In order to minimize potential risks, individual categories have specified events/factors which may have a negative impact on the patient’s safety.

### 2.2. The Course of the Research Procedure

The methodology of the research procedure used in the study, based on the newly proposed PM model as the primary research tool, includes the following stages:

1. Collection, review and analysis of the literature data regarding the subject; identification of events/risk factors
2. Determination of the probability (P) (frequency) of an event/factor on a discrete scale from 1 to 5
3. Determination of the Severity of the Event (SR)
4. Indication of the directions for improvement of the events which have occurred.

According to scientific literature, there is a certain group of reasons (events or factors) which contribute to deviations from the assumed requirements [2,4,10,12,13,14,15,19,20,21]. Publications focused on the identification of the hazard risk and exposure to the risk of infection allow to distinguish several basic, general determinants which cause patient safety breaches. They are primarily found in the areas of human, administrative and management errors as well as errors resulting from the lack of proper education and culture.

Therefore, they can be also divided into:

1—reasons related to the hospitalized patient (e.g., age, chronic diseases, endogenous infections).

2—organizational reasons, indirectly related to the hospital’s organizational culture, processes, knowledge transfer, internal employee communication (e.g., control of procedures or training not adapted to the specifics of the ward and the needs of the staff).

3—explicit reasons resulting from direct contact with the patient (e.g., medical workers do not properly observe hand hygiene).

4—hidden reasons, resulting from the behavior of medical workers and their culture, from decisions which impact the established policies, the operating and medical procedures, and proper management of resources.

5—reasons of a technical nature resulting from defects and faults related to devices, materials, equipment and external resources (e.g., sterilization room breakdowns, air conditioning failures, elevator breakdowns). Examples of events/factors that were assigned to a specific problem category associated with the infrastructure and management of a medical facility as well as connected with the hospitalized patient are listed in Table 1.

For the analysis of hazards and exposure to the risk of nosocomial (HAI) and *C. difficile* infections in the studied hospital, it was decided to use the novel Proposed Model (PM) described earlier. It was used in the research because it facilitates the gathering of information regarding the possible causes of the analyzed phenomena and enables the identification of areas from the field of management of the entire medical facility which require particular improvement, control or change, taking into account the potential risk effects. In the first part, after the initial analysis of risk factors, the probability (P) (frequency) of the occurrence of a given event/factor was determined in the designated hospital risk categories. The given probability was assigned an appropriate point value (discrete scale from 1 to 5) (Table 2). In the next stage, the severity of the event (SR) was calculated, defined as the arithmetic mean of its categories of events (W) and event mitigation (Z), i.e., risk minimization activities (Table 3). The range for the mitigation of the event (Z) is on a discrete scale from 5 to 1. Thus, obtained numerical values saved in a spreadsheet enable the calculation and indication of the relative risk (R) (relative hazard) of the occurrence of a given event. Its interpretation is carried out according to the values contained in Table 4.

In the PM model it was assumed that in the final stage the relative exposure to the risk of nosocomial infections (R) is expressed as a percentage [%] as the product of the P and SR values. After calculating the value of the risk for a given hazard, we proceed to the assessment of this risk in order to determine its potential danger or hazard it can pose to a hospitalized patient (Table 4).

The assessment of the Risk (R) value for a potential hazard or exposure was determined on the basis of a risk matrix with assigned ranges on a scale from 1 to 25 points (according to the matrix) and the corresponding scale from 0 to 100%. A detailed list of the above values is presented in Table 4.

Based on the calculations and the obtained results, it is possible to indicate directions of improvement for all potential events, to minimize their frequency and, at the same time, to obtain adequate information regarding the directions of actions to be taken in case of a crisis situation associated with the occurrence of a given phenomenon. The selection of such a model was also justified by the fact that, despite the time and labor involved in its application, the literature regarding the subject constantly confirms the usefulness of searching for all causes of nosocomial infections, adverse events, including infections associated with *C. difficile*, and methods of their elimination.

In the PM conceptual model, we used research material from one of the largest city hospitals located in the central part of Poland. The material concerning the research part for *C. difficile* infections originated from the Intensive Care and Anesthesiology Unit (ICAU) during the years 2018–2020. The source of this material was an analysis of periodic nosocomial infections’ cards, including emergency pathogen infections, outbreak reports and patient disease cards.

The Intensive Care and Anesthesiology Unit selected by the Team is one of the primary wards present in every hospital. This is where critical patients are being treated, and it differs from other wards mainly in that it admits patients in a very severe condition which requires continuous observation of vital functions and respiratory, circulatory and/or digestive system support. This ward is usually equipped with multiple complicated medical devices, which monitor and support the patient’s vital functions. A specialized team of nurses, doctors, EMTs, physiotherapists and technical staff should work at the ward. The ward works with other hospital wards, laboratory, consults on the treatment, diagnostics, nutrition and rehabilitation.

The specifics of intensive care and anesthesiology units, resulting from the provision of medical services to patients with life-threatening conditions, who need highly specialized medical care and ventilator therapy requires ensuring an appropriate staffing level of medical personnel. The number of personnel working at intensive care units is much higher than the average per 1 bed at other hospital wards, however under the conditions of the specific unit under analysis it does not meet the optimum requirement for the number of performed medical services and for the workload of individual beds. Many times, problems occur with providing basic duty personnel. Staffing shortages are supplemented with contracts signed for single shifts [2]. External employees who perform tasks frequently do not demonstrate sufficient knowledge of the work organization procedures and principles in force at the facility. During an occurrence of critical moments, when rapid and precise decisions have to be made this introduces communication chaos, which may result in adverse events, frequently occurring as a result of problems with the hand hygiene principles and with hand hygiene process control [10,15].

In terms of the analysis of the entire infrastructure and hospital management, the material concerning the research part was obtained from individual hospital wards and administrative departments. People designated for the study responsible for the supervision over the correct functioning of individual wards and the entire medical facility were asked to fill out individual problem domains in order to determine the hazard risk and exposure to the risk of infection with multi-drug-resistant microorganisms associated with the provision of health services.

People responsible for providing answers and for making assessments (according to a point scale specified in the model) of existing events and of risk have appropriate experience and qualifications. These people are responsible for the establishing of decision making procedures to be applied during critical situations, for identification of events, for documenting and for verifying the decisions that were made, which apply both to the functioning the entire medical facility and the medical procedures at individual ward.

### 2.3. Characteristics of the Medical Facility

The research with the use of the PM model was carried out in a hospital that provides tertiary care. Data regarding the characteristics of the analyzed medical facility with 32 wards is presented in Table 5.

The largest wards (with the highest number of medical staff) include: Intensive Care and Anesthesiology (124 people), Hospital Emergency Department (75 people) and the Department of Teleradiotherapy (73 people), whereas the smallest ones are the Department of Hygiene and Epidemiology (7 people) and the Department of Hematology and Chemotherapy (7 people).

## 3. Results

The number of hospitalized patients in particular years (admitted to hospital for ≥48 h from 1 January 2018 to 31 December 2020), the total number of all nosocomial infections and the number of *C. difficile* cases in the entire hospital are presented in Table 6.

In the hospital under analysis the incidence (HAI) per 100 admitted patients amounted from 2.04 to 2.50% in the years 2018–2020. The number of reported *C. difficile* cases among all nosocomial infections in individual years ranged from 6.3 to 11.3%, i.e., 11.3% in 2018, 6.3% in 2019, and 11.0% in 2020. The highest number of infections was found in the following departments: Internal Medicine, Intensive Care and Anesthesiology, Stroke with Early Neurological Rehabilitation, Vascular Surgery, General and Oncology, Cardiology, Neurosurgery and Nervous System Neoplasms, the least infections were found, among others, in the departments of: Rheumatology and Systemic Connective Tissue Diseases and Gynecology and Oncology Surgery, and the total absence of cases of infections caused by *C. difficile* was, among others, in the departments of: Laryngology Oncology, Interventional Pediatrics and Neurology. The chain of epidemic infections caused by particular pathogenic microorganisms may occur throughout the entire hospital infrastructure. Therefore, in the proposed PM model, each of the hazard categories (Table 1) was assessed in the framework of an individual and comprehensive assessment of the hazard and exposure to the risk of HAI. Due to the fact that in the study we focused on nosocomial infections associated with the *C. difficile* bacterium occurring in individual hospital wards, in the final part of the analysis we took the following categories into particular consideration—Assessment of a given ward (factors—human, technical), Assessment of technical facilities (hospital failure rates), Assessment of organizational factors, Assessment of explicit factors resulting from direct contact with the patient, due to the fact that they have a notable impact on *C. difficile* infections in the first place after the admission of a hospitalized patient. The Intensive Care and Anesthesiology Unit was selected from among 32 hospital wards. This ward is characterized by an increased number of *C. difficile* infections among hospitalized patients (on average 23 cases per year) and a very high total number of infections in the examined medical facility (on average 29.3% per 100 admitted patients). The detailed specifics of infections in the selected ward are presented in Table 6.

The selected ward is characterized not only by a high number of nosocomial infections and *C. difficile* infections among hospitalized patients, but also by the specificity of treatment, performed medical procedures, the condition and health of the patients admitted for treatment, the number of employed medical and auxiliary staff and management organization, which may contribute to adverse events, including the spread of biological pathogens. That is why based on the new PM model an attempt was made to indicate events that could be eliminated and events with a deciding impact on the potential of HAI occurrence. For research purposes (and for comparative purposes in the future), the last period of operation of the selected ward, i.e., the year 2020, was taken into account. In order to determine the risk characteristics of the ward, Table 7 summarize the results of all components for 2020, which were developed according to the PM model in a spreadsheet (Excel) with events/factors assigned to a specific category of hazards related to a medical facility (Table 1), and assessed parameters characterizing the probability (P) of a given event and the severity of the SR event (Table 3).

**Figure 3:**
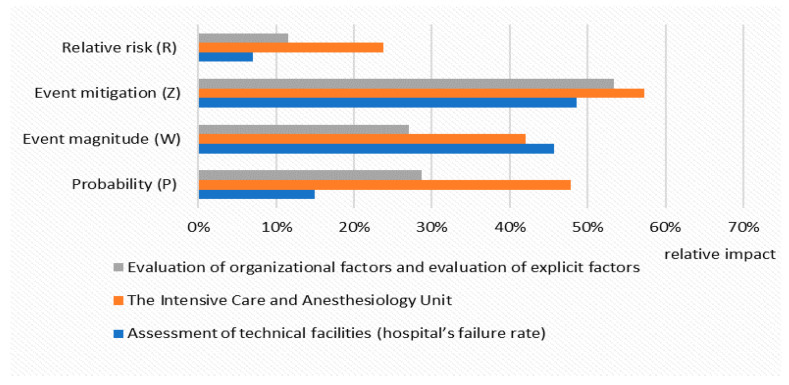
Relative risk (R) of *C. difficile* infections for hospitalized patient in selected risk categories, with specific parameters included in the PM model.

On the basis of the calculations and results obtained in the PM model (Excel spreadsheet), the relative risk (R) of *C. difficile* infections expressed as a percentage [%] was assessed. Figure 3 presents the relative risk of *C. difficile* infections for a hospitalized patient staying in the Intensive Care and Anesthesiology Unit, for the technical facilities (hospital’s failures) and for organizational and explicit factors resulting from direct contact with the patient, with specific parameters characterizing the event’s probability (P) and the event’s magnitude (W).

After an analysis of the individual components of events/factors occurring in individual categories, the gravest events with the highest relative risk (R) of their occurrence which affect the hospitalized patient’s safety were presented in Table 8, Table 9 and Table 10.

**Figure 4:**
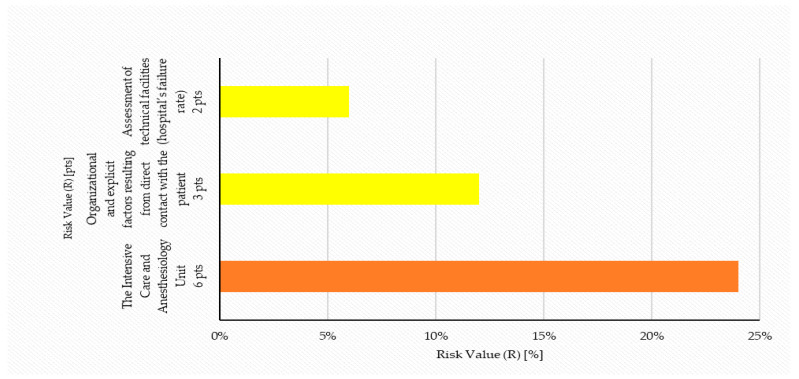
Potential risk or exposure for the hospitalized patient that may be caused the Intensive Care and Anesthesiology Unit, technical facilities (hospital’s failure rate) as well as organizational factors and explicit factors resulting from direct contact with the patient.

The calculations obtained this way were used for the final compilation, the analysis of hazards occurring in the selected categories and for the graphical interpretation of the final results. After calculating the risk value (R) for a given hazard, a verbal specification was written for the potential hazard or exposure (risk) that may be posed by the analyzed category for the hospitalized patient (Table 11 and Figure 4). The assessment of the Risk (R) value was determined on the basis of a risk matrix with assigned ranges on a scale from 1 to 25 points (according to the matrix) and the corresponding scale from 0 to 100%.

## 4. Discussion

The primary duty of each hospital is to ensure the safety of hospitalized patients. This task is achieved by risk management, which must incorporate systemic measures integrating various processes: medical, management and supportive. Appropriate supporting models and tools are helpful in risk management. It was successfully demonstrated that our PM model is a tool that supports risk management. With the use of PM, we have shown that the dynamics of the spread of nosocomial infections can be controlled and the critical level of infections can be minimized by demonstrating the events or factors contributing to the occurrence of adverse events.

The study based on the PM model revealed the existence of numerous events and/or factors that carry a high level of risk and a high possibility of infecting a hospitalized patient. In the category of technical facilities (hospital failure rate), it was found, inter alia, that the risks of ventilation and air conditioning breakdown are at a level of 8% and 10%, respectively. It can be said that these risks are at a low level, however taking into account the level of risk at the chosen ward—Intensive Therapy and Anesthesiology Unit, in patient rooms, where untimely/improper servicing of gravity ventilation, of supply and exhaust ventilation and of air conditioning system are already at the risk level as high as 50%, it can be safely said that failures of this type can create (pose) a significant risk of infection with the *C. difficile* bacterium or other pathogen, due to the lack of internal air exchange. The hospital environment, in the very specifics of its functioning, accumulates a much higher concentration of pathogens than an average building and external environment, as most microorganisms move in air currents, with heating, ventilation and air conditioning (HVAC) devices being very susceptible to the accumulation of large amounts of pathogens, and even possibly serving as niches for their proliferation and multiplication. Additionally, those devices play a very important role in the use of medical equipment, which often requires perfect control of physical air quality, because they are sensitive to temperature and humidity fluctuations, especially in the Intensive Care and Anesthesiology Unit [19,22]. Numerous scientific studies conducted in medical institutions confirm the positive or negative impact of HVAC on the vital parameters of the hospitalized patient [22,23,24,25]. The study described by Lenzer & Rupprecht demonstrated that the impact of HVAC on the health of the hospitalized patients had an important role in the area of improved regeneration, manifested by increased improvement of vital signs, reduced heart load, faster recovery and greater physical activity. All these factors contributed to shorter hospital stays, reduced mortality and financial savings for the hospital [19].

Complete systems for generation of medical air, vacuum pumps, compressed gas pressure regulation systems are separate medical devices, which should meet the requirements of the PN-EN ISO 7396-1:2016 harmonized standard. The final element of the medical gas system are the gas outlet points. The most basic solution is placing them directly in the wall, but they are frequently installed in supply units, such as bed head units in patient rooms or ceiling columns in ICU or operating rooms. Antiquated designs for distribution and consumption of gases do not ensure safety of use, and thus may constitute a significant risk to the success of therapy.

Even though at the Intensive Care and Anesthesiology Unit the lowest risk levels (R) were established for the number of patients (R = 15%), problems with medical gases supply (R = 10%) and equipment age (R = 14%), these events should not be minimized, since the better the control of the immediate environment of the patient and the newer (more modern) equipment related to maintaining the patient alive, the lower the risk of HAI and the lower number of potential deaths or complications. At the same time, at this ward in 2020 a total of 12 HAI deaths were established, that is why in accordance with the criteria of the PM model all events/factors which may be eliminated or significantly restricted have to be found.

Relative risk (R) for the most blatant events/factors in the category of technical facilities (hospital failure frequency) and for the organizational factors and overt factors resulting from direct contacts with the patient was significantly lower than directly at the Intensive Care and Anesthesiology Unit and was respectively in the range of 8 to 29% and 8 to 34%. These two categories of events/factors were established on the scale of potential risk (Table 11) as low, but significant. Thus, the highest risk with an impact on the hospitalized patient occurs directly at a specific ward, since the remaining categories of events may be very similar within the area of the entire hospital.

Highly invasive medical procedures pose a direct hazard to the patient who is prone to infection from both exogenous and endogenous flora, which constitute significant pathogenic factors for immunosuppressive patients with chronic diseases causing organ dysfunction [26]. The use of disposable equipment reduces the risk of contamination and transmission of pathogens between patients and hinders the formation of biofilms. Controlling the sterilization and disinfection processes of the used instruments and devices is crucial to ensure safe hospitalization. Maintenance of cleanliness in the ward and detailed implementation of the rules related to cleaning and disinfection of surfaces, especially those in the area closest to the patient or in the area of direct exposure play an equally important role [26]. The selection of appropriate disinfecting preparations with confirmed effectiveness allows minimizing the presence of spores and other microorganisms in the environment. Treatment of patients with cardiopulmonary insufficiency, suspected septic shock, on dialysis or with severe injuries goes hand in hand with the administration of antibiotics. The number of HAI detected in intensive care units significantly exceeds the average value calculated for the entire medical entity. Analysis of a microbiological map defining the microorganisms causing the greatest number of infections indicates that intensive care units are characterized by a highest percentage of multi-drug-resistant strains, which require treatment with antibiotics and chemotherapeutic agents, often referred to as drugs of the last resort [8,27]. Broad-spectrum antibiotic therapy contributes to the selection of resistant strains, leads to the elimination of natural microbiota, which creates space for pathogens, especially in relation to *C. difficile* [2]. It should be emphasized that vancomycin is most often used in the treatment of full-blown CDI, which in turn leads to the occurrence of vancomycin-resistant *Enterococcus* (VRE) in the patient, hence each action taken has specific effects impacting the patient’s medical condition.

In addition, the PM model enabled identification of phenomena that do not attract attention on a daily basis, and are very important in the epidemiological chain. In the category of human and organizational factors, it was demonstrated that there is a risk of infringing on the patient’s safety due to the lack of a clearly presented organizational structure of the hospital (risk at a level of 31%), resulting in visible chaos, lack of effective hospital management and risk management. The model demonstrated that new rules and regulations are insufficiently implemented on an ongoing basis, medical procedures are not always improved or controlled, and the effectiveness of infection prevention measures is rarely assessed. Moreover, according to the proposed PM model, a preliminary analysis of the results (not presented herein) indicates that in the selected (worst) wards there are several similar hazards, which may be related not only to the lack of effective management, but also to the presence of *C. difficile* infections. These hazards usually include: a large amount of unnecessary medical equipment in patient rooms, a shortage of equipment with an antibacterial coating in wards, non-frequent servicing of HVAC, a shortage of doctors and nurses, and fairly frequent failures of the system linking the system for monitoring patient parameters with the nurses’ station. These factors have a notable impact on patient safety, and at the same time show gaps in the system of functioning of risk management in the hospital [10]. All risk related activities should be closely related to the goals and tasks of the hospital and must be included in the framework of the process in which there is an effective and properly integrated tool enabling a reliable hazard analysis.

By using the PM model in other departments of the hospital under analysis, the following significant risk factors were found: an increased average age of hospitalized patients and the severe overall condition of the patients, which often led to a prolonged hospital stay for almost two months on average. Although the number of hospitalizations at the Department of Internal Medicine (DIM) was the highest, the incidence per 100 admitted patients and the average length of stay in hospital were the lowest, whereas in the Intensive Care and Anesthesiology Unit, in which the number of hospitalizations was the lowest, there was the highest incidence rate and the longest average duration of a patient’s stay in hospital. These values indicate that the incidence is not related to the number of hospitalized patients, but results from the nature of a given ward and the health condition of the admitted patients. In both analyzed departments, a fairly similar number of cases of *C. difficile* infections was also found in 2018–2020 (ICAU—25, DIM—20), and the number of employed medical personnel differed significantly (ICAU—124 people, DIM—27 people). The above observations are confirmed in the literature [8,27,28,29].

Some of the risk factors can be reduced or eliminated (e.g., unnecessary equipment in patient wards, deficiency of antibacterial coatings on surfaces of medical equipment (beds, door handles, handgrips), and inadequately trained cleaning staff), which may contribute to minimization of hospital infections, including infections associated with *C. difficile* and, most importantly, improve patient safety and shorten their stay at the ward. But in order to achieve such results, particular attention should be paid not only to components such as knowledge and skills, but also to clear communication between hospital employees (risk 28%), which may result in an unpleasant atmosphere, adverse events in hospitalized patients, rotation of medical staff (risk 17%) as well as teamwork and management culture. In the case of HAI transmission, the epidemic chain is most often caused by neglect and failure to follow basic procedures at a given ward. Additional factors include insufficient number of training session, training courses not tailored to the specifics of the ward and needs of the staff, improper communication between medical workers, rarely performed evaluation of the effectiveness of measures to prevent infections, e.g., compliance with hand hygiene procedures [13,26]. Very helpful measures that can be implemented in such cases include: regular training of all medical workers, compliance with hand hygiene, maintenance of cleanliness in patient rooms, etc. In many cases, these elements are supervised by infection prevention teams and epidemiological nurses, the number of which is usually insufficient in hospitals (6 people in the studied case). Additionally, the routine of performed activities in relation to the patient contributes to the possibility of transmission of nosocomial infections.

In a similar way as described above, each hospital department should be characterized with the use of the proposed PM model, since the obtained results confirm that it is not possible to assess the risk for the entire medical facility on the basis of one or two wards. Although some potential events may impact the entire hospital (e.g., interruptions in telephone communication, sterilization room breakdowns, gas outages), the vast majority of factors are related to individual wards and the condition of the patient admitted to the ward. Persons responsible for the functioning of a given ward is the doctor—head of the ward or chief physician. These persons, along with the ward nurse (coordinating nurse) supervise the organization and work of a given ward. The assessment of organizational factors and factors affecting patient safety also plays a very important role in the entire medical facility.

## 5. Conclusions

This PM model for predicting the hazard risk and exposure to the risk of nosocomial infections has proven successful at the Intensive Care and Anesthesiology Unit in the examined medical facility. More research is needed to determine if it can be used to prevent hospital-induced risks and infections in patients and to reduce hospital costs in other hospital settings and wards. The precise determination of the probability (frequency) of a given event (P) and the severity of the event (SR) is a very important element in the entire model. A person related to the management of the medical facility should be responsible for the analysis and processing of the data, and this person, in cooperation with the head physicians, should be able to identify all the problems prevailing in a given ward. All comparisons of possible and past crisis situations which occurred in previous years both at the wards and in the entire medical facility are also helpful. Collection of this data is necessary in order to create a detailed spreadsheet, which may differ between individual wards and also between individual medical facilities. Accurate data on hospitalized patients (hospital admission card and discharge from the hospital) are the source of knowledge regarding potential nosocomial infections and treatment costs assigned to individual wards.

In our research conducted with the use of the novel PM model, it was found that each ward has its own specific problems that can be eliminated only with knowledge of potential events that may occur in a given department. The obtained results can be used for consultation with the people responsible for the management of individual wards as well as the entire medical facility. They are also the basis for the development of detailed plans to prevent the occurrence of a given event and action plans when a given event occurs.

## Figures and Tables

**Table 1:** Examples of events/factors related to the infrastructure and management of a medical facility.

**Assessment of technical facilities (hospital’s failure rate)**		
failure of the room disinfection system	incorrect laboratory results	air conditioning failure
improper disinfection of the sickroom	untimely laboratory results	staff shortages (sudden illness, etc.)
improper decontamination of reusable equipment	inadequately trained cleaning staff	a work accident of hospital staff
		microbial contamination of water
inadequate quality of cleaning of the hospital environment	antibacterial covers (bed, door handles, handles, etc.)	failure due to inadequate infrastructure
		failure of the fire suppression system
failure of medical equipment	breakdown at the boiler room (or district heating)	internal fire
shortage of personal protective equipment (including gloves and aprons)	no access to hot water	gas supply outages
	no access to cold water	power supply outages
shortage of disposable equipment	breakdown at the laundry room	interruptions in internet access
shortage of disposable materials in the ward	flooding with water	failure as a result of renovations
problems with the supply of medical gases	sewerage failure	failure of convenient access for ambulances
sterilization room breakdown	elevator breakdown	insufficient parking area for patients
crash of the patient registration system	ventilation breakdown	insufficient parking area for hospital staff
lack of a medicine in the hospital pharmacy	interruptions in telephone communication	failure of the electrical network
**Evaluation of organizational factors and evaluation of explicit factors resulting from direct contact with the patient**		
organizational structure, whether it is clearly presented	whether the procedures are constantly improved	low awareness of the staff about nosocomial infections
rules and/or new regulations, whether they are implemented on a regular basis	control of HH (hand hygiene) procedures	medical personnel do not follow proper hand hygiene
general and vague procedures	communication between the staff	medical personnel do not follow proper hospital hygiene
limited access to procedures		
whether there is an assessment of the effectiveness of infection prevention measures	responsibilities of team members in the departments are not clearly defined	the routine of the work performed
		professional burnout
no method of remedial action	too small number of training courses	funds for prophylaxis
general control of procedures	non-compliance with procedures such as HH	training not adapted to the specifics of the department and needs of the staff
**Assessment of a given department: human and technical factors**		
shortage of doctors per ward/number of patients per doctor	gravity ventilation in patient rooms	servicing of air conditioning system
shortage of nurses for a given ward	servicing of gravity ventilation system in patient rooms	operational bell system for connecting patients with the nurses’ station
number of patients per nurse		
number of patients in the ward/bed occupancy	supply and exhaust ventilation in patient rooms	shortage of medical devices in the ward
toilet in patient rooms	air conditioning system in patient rooms	epidemic in the ward
bathroom in patient rooms	servicing of supply and exhaust ventilation system	epidemic outbreak in the ward
invasive procedures are performed	amount and quality of equipment in patient rooms	most common pathogens
age of medical equipment		
**Assessment of risk factors related to the patient’s condition upon admission to the unit (and during hospital stay), which may adversely affect the safety of the patient, contact patients, staff and the entire hospital environment**		
age	cancer/radiotherapy/chemotherapy/steroidotherapy	renal failure requiring dialysis
active acute infection (e.g., pneumonia, invasive infection, urinary tract infection (UTI), etc.)	antibiotic therapy up to three months before hospitalization	open injury/internal injury/multi-organ injury
chronic infections or carriage (e.g., hepatitis B/hepatitis C/tuberculosis/HIV/AIDS/Lyme disease)	transfusion of blood products up to six months before hospitalization	artificial pathways (urinary catheter, stoma, vascular catheter, tracheal tube, etc.)
skin lesions/hygiene negligence/urinary incontinence/fecal incontinence	chronic diseases (e.g., diabetes, heart failure, kidney failure etc.)	the patient is unconscious/intubated/after SCA/with a tracheostomy tube
addiction (alcohol, nicotine, pharmaceuticals, drugs, etc.)	active acute infection, vector of an alarm pathogen	transfer from another hospital or hospitalization in the last six months
previous surgical procedures/invasive tests (endoscopies, injections, dental procedures, etc.) performed in the last 3 months	permanent medications (immunomodulators, anticoagulants, non-steroidal anti-inflammatory drugs, proton pump inhibitors, insulin, etc.)	contact with an infectious patient/carrier of an alarm microorganism in the last 3 months
		recurrent inflammatory processes (e.g., adnexitis, sinusitis, recurrent boils, etc.)
**Assessment of hospital determinants which induce the lowering of safety level of the patient** **undergoing hospitalization**		
type of ward where the patient is staying	patient connected to a drip-bag	Diarrhea
anticipated hospitalization time/length of hospitalization	toilet in the patient room/shared toilet for patients outside the patient rooms	whether PPI (proton pump inhibitors) are used
lying/walking patient	patient undergoing dialysis	dose of antibiotic
antibiotics used	blood transfusion	number of days of the antibiotic
whether a catheter is used	breathing problems	peripheral puncture
patient room	patient with allergies	urinary catheter
whether the patient uses disinfectants	whether the patient is visited by other patients	whether the patient uses the social rooms/kettle in the corridor
whether the patient is visited by the family	patient with a stoma	Respirator
allergy to selected medications	special diet	probe (feeding/decompression)
central catheter	parenteral nutrition	temperature over 38 °C
bedsores	patient dehydration	frequency of the patient’s contact with the biological agent/patient with CDI or suspected of CDI/room

Source: Own study.

**Table 2:** Determination of accident probability (P) (frequency) for hazards and exposure to risk of *C. difficile* and HAI infections.

Value	Characteristics
1	Very rare, but occurring in reality
2	Rare
3	Moderately frequent
4	Frequent
5	Very common, almost certain

Source: Own study.

**Table 3:** Parameters characterizing the probability (P) (frequency) of an event occurrence, the magnitude of the event (W) and event mitigation (Z), and the risk (R).

**Probability (P)** (frequency)	**Event Severity (SR)**						**Risk (R)**
	**Event Magnitude (W)**			**Event Mitigation (Z)**			
	Human life and health	Fixed assets	Impact on facility management	Readiness	Internal resources of the organization	External resources	
P that it will happen	Possible death or injury	Physical loss and damage	Interruptions in the provision of services	Plan in case of an incident	Time, efficiency, other	Staff and supplies	Relative risk expressed in [%]
Discrete scale from 1 to 5	Discrete scale from 1 to 5			Discrete scale from 5 to 1			Scale from 0 to 100% (1–25 points)

Source: Own study.

**Table 4:** Numerical values and verbal interpretation for a given Risk scale (R).

R Low (Yet Significant)		R Medium (Very Significant)		R High Risk (Unacceptable)	
R ≤ 0.20 (Up to 20%)		0.21 < R ≤ 0.60 0.21 (21–60%)		R > 0.60 (>60%)	
pts	[%]	pts	[%]	pts	[%]
1	0	6	21	16	63
2	4	7	25	17	67
3	8	8	29	18	71
4	13	9	33	19	75
5	17	10	38	20	79
		11	42	21	83
		12	46	22	88
		13	50	23	92
		14	54	24	96
		15	58	25	100

Source: Own study.

**Table 5:** Characteristics of the hospital under analysis.

Characteristics of the Unit	Numerical Value
Number of beds	950
Number of wards	32
Number of employed medical staff (excluding administration)	1349
Number of medical staff per bed	1.42
Number of doctors	266
Number of nurses	735
Number of other medical staff	348
Number of epidemiological nurses	6
Number of employees in the infections team	5 nurses and 1 doctor working part-time
Number of people in the hospital administration (excluding HR, payroll, economic, financial, organizational and legal, public procurement, medical controlling, internal control, transport, administrative and economic departments)	235

**Table 6:** Characteristics of the hospital and of the Intensive Care and Anesthesiology Unit (ICAU) in 2018–2020.

Parameter under Analysis	Year		
	2018	2019	2020
Number of the treated/hospitalized	67.064	67.466	58.854
Number of *C. difficile* cases	86	91	68
Number of tests for *C. difficile*	613	637	499
Mean length of stay for hospitalization with diagnosis of *C. difficile* (24 h)	14.61	20.30	12.57
Total number of HAI	762	671	621
Incidence (HAI) per 100 admitted patients [%]	2.35	2.04	2.50
Overall number of bacteriological tests	21.204	25.065	21.118
**Intensive Care and Anesthesiology Unit**			
Number of *C. difficile* infections, toxin A/B	25	27	18
Number of hospitalizations	420	406	403
Number of person-days of hospitalization	6092	6001	6264
Number of nosocomial HAI infections	143	114	104
Incidence (HAI) per 100 admitted patients [%]	34.05	28.08	25.8
Incidence (HAI) per 1000 person-days of hospitalization	23.47	19.00	16.6

**Table 7:** Characteristics of the Intensive Care and Anesthesiology Unit and patients with HAI and with *C. difficile* infection in 2020.

Parameter under Analysis	ICAU
**Characteristics of the Unit and of patients with HAI**	
Number of beds per ward	19
Number of doctors	15
Number of nurses	91
Number of other employees	18
Number of treated/hospitalized	403
Number of HAI	104
Pathogens most often isolated	*Klebsiella pneumoniae*, *Acinetobacter baumannii complex*, *Escherichia coli*, *Staphylococcus aureus*
The most common clinical form of HAI	Blood infection [49.0%]
Average hospital stay time of a patient with HAI	52 days
Number of deaths due to HAI	12
**Characteristics of patients with *C. difficile***	
Average age	61 years
Dominant gender of patients	54 [%] women
Average hospital stay time	58 days
The most frequent primary diagnosis	Circulatory and respiratory failure
Number of deaths due to *C. difficile*	4
Number of *C. difficile* infections, toxin A/B	18

**Table 8:** Relative risk (R) for the gravest events/factors in the category of a given ward—the Intensive Care and Anesthesiology Unit (human and technical factors).

Most Dangerous Event/Factor	Level (R) [%]
shortage of doctors	29
shortage of nurses	31
number of patients in the ward	15
problems with supply of medical gases	10
antibacterial covers (bed, door handles, handles, etc.)	54 ^1^
amount of equipment in patient rooms	54 ^1^
age of medical equipment	14
gravity ventilation system service in patient rooms	50 ^1^
gravity ventilation in patient rooms	27
supply and exhaust ventilation system service	50 ^1^
supply and exhaust ventilation in patient rooms	41
air conditioning system service	50 ^1^
air conditioning system in patient rooms	41
operational bell system for connecting the patient with the nurses’ station	58 ^2^

^1^ values close to high risk (not acceptable); ^2^ high risk (unacceptable).

**Table 9:** Relative risk (R) for the gravest events/factors in the category of technical facilities (hospital’s failure rates).

Most Dangerous Event/Factor	Level (R) [%]
interruptions in telephone communication	10
air conditioning failure	10
ventilation breakdown	8
failure of the electrical network	13
failure of the fire system	11
crash of the patient registration system	21
incorrect laboratory results	16
untimely laboratory results	25
an accident at work of hospital staff	14
staff shortages (sudden illness, etc.)	29
inadequately trained cleaning staff	11
equipment failure of medical	13

**Table 10:** Relative risk (R) for the gravest events/factors in the category of organizational and explicit factors resulting from direct contact with the patient.

Most Dangerous Event/Factor	Level (R) [%]
medical staff rotation	17
organizational structure, whether it is clearly presented	31
rules and/or new regulations, whether they are implemented on a regular basis	25
whether the procedures are constantly improved	28
whether there is an assessment of the effectiveness of infection prevention measures	34
funds for prophylaxis	8
insufficient number of trainings	9
trainings not specifically tailored to the specifics of the department, staff needs	9
communication of the staff	28
professional burnout	25
the routine of the work performed	11

**Table 11:** Potential risk or exposure for the hospitalized patient that may be caused by the Intensive Care and Anesthesiology Unit, technical facilities (hospital’s failure rate) as well as organizational and explicit factors resulting from direct contact with the patient.

Category Analyzed	Risk Value (R)		The Scale of the Potential Risk
The Intensive Care and Anesthesiology Unit	R = 6 pts	24 [%]	Medium (very significant)
Organizational and explicit factors resulting from direct contact with the patient	R = 3 pts	12 [%]	Low (but significant)
Assessment of technical facilities (hospital’s failure rate)	R = 2 pts	7 [%]	Low (but significant)

## Data Availability

The data presented in this study are available on request from the corresponding author.
